# The anterior cingulate cortex contributes to the analgesic rather than the anxiolytic effects of duloxetine in chronic pain-induced anxiety

**DOI:** 10.3389/fnins.2022.992130

**Published:** 2022-11-24

**Authors:** Chenglin Li, Kaiji Ni, Meiru Qi, Jie Li, Kexin Yang, Yanli Luo

**Affiliations:** ^1^Department of Psychological Medicine, Renji Hospital, Shanghai Jiao Tong University School of Medicine, Shanghai, China; ^2^Shanghai Mental Health Center, Shanghai Jiao Tong University School of Medicine, Shanghai, China; ^3^School of Life Sciences and Technology, ShanghaiTech University, Shanghai, China

**Keywords:** duloxetine, chronic pain, anxiety, anterior cingulate cortex, fiber photometry

## Abstract

Mood disorders, such as anxiety and depression, are commonly found in people suffering from chronic pain. Serotonin–norepinephrine reuptake inhibitors (SNRIs) are potential in alleviating chronic pain and are the first-line option for anxiety disorder. The anterior cingulate cortex (ACC) plays a vital role in chronic pain-induced anxiety, but its role in the therapeutic effects of SNRIs remains largely unclear. We used complete Freund’s adjuvant (CFA) in this current study to induce chronic inflammatory pain. Von Frey test was used to measure the mechanical withdrawal threshold. The elevated plus maze test (EPM) and the novelty-suppressed feeding test (NSF) were used to measure anxiety-like behaviors. Twenty-one days after the modeling, anxiety-like behaviors were successfully induced in CFA mice, and a 3-day intraperitoneal injection of duloxetine attenuated such behaviors. While, mechanical hyperalgesia was also improved. Then, we locally infused duloxetine in ACC for 3 days only to find out its analgesic effect in CFA mice. Furthermore, we used fiber photometry to discover decreased glutamatergic excitability and enhanced serotonin concentration in ACC after intraperitoneal injection of duloxetine. Overall, this study proposed a potential mechanism for the analgesic effect of duloxetine and shed light on further studies on the mechanism of its anxiolytic effect in chronic pain-induced anxiety.

## Introduction

Pain is an unpleasant sensory and emotional experience related to actual or potential tissue damage (Pain, 1979). Mood disorders such as depression and anxiety disorder are commonly developed in people suffering from chronic pain, which is shown in multiple epidemiological studies (Tsang et al., 2008; Hilderink et al., 2012; Velly and Mohi, 2018). The burden of pain and anxiety is high from physiological, psychological, and societal perspectives. Moreover, a study indicated that pain in multiple locations is more likely to develop the first onset of anxiety disorder (Gerrits et al., 2014). Overall, treatment strategies containing both analgesic and anxiolytic effects should be established for patients with chronic pain.

Consequently, preclinical studies focused on such issues. They managed to develop anxiety-like behavior in mice with persistent inflammatory or neuropathic pain induced by the injection of complete Freund’s adjuvant (CFA) or by spared nerve injury (SNI) (Sellmeijer et al., 2018; Liang et al., 2020). Furthermore, brain regions and neural circuits related to pain-induced anxiety have been studied, among which the most frequently mentioned is the anterior cingulate cortex (ACC) (Zhuo, 2016). Clinically, the imaging study showed the involvement of ACC in pain processing, and preclinical evidence demonstrated that the activation of ACC neurons is associated with pain-like behavior. In contrast, the inhibition of these neurons attenuated such behavior (Barthas et al., 2015). As for pain-induced anxiety, presynaptic long-term potentiation (pre-LTP) in ACC was found in mice with chronic pain-induced stress, while eliminating it had an anxiolytic effect in CFA and SNI models (Koga et al., 2015). Another study also discovered ACC hyperactivity in chronic pain-induced anxiety, more shown explicitly by the increased excitatory postsynaptic transmission and increased involvement of N-methyl-D-aspartate (NMDA) receptors (Sellmeijer et al., 2018). These results suggested not only vital role of ACC in pain-induced anxiety but also shed light on a potential therapeutic target of a particular medicine.

As a first-line of treatment for generalized anxiety disorder, duloxetine, one of the serotonin–norepinephrine reuptake inhibitors (SNRIs), has displayed its potential in alleviating chronic pain and pain-induced anxiety (Lunn et al., 2014). However, the mechanisms of effects of duloxetine on both symptoms remain unclear, hindering the broader usage of this drug. Serotonin is related to anxiety disorder and chronic pain (Marcinkiewcz et al., 2016; Paredes et al., 2019). The excitability of glutamatergic neurons in ACC is significantly reduced after the local injection of serotonin, and such reduction can be reversed by a 5-HT1A receptor antagonist (Tian et al., 2017). Above all, it is essential to verify the potential therapeutic effect of duloxetine on chronic pain-induced anxiety and its mechanism by upregulating the 5-HT1A receptor and thus reducing the excitability of glutamatergic neurons.

In this study, we discovered the intraperitoneal effect of duloxetine on anxiety and pain in mice with chronic inflammatory pain through behavioral tests on mechanical hyperalgesia and anxiety-like behaviors. As an assumptive target region, we locally injected duloxetine into ACC to confirm whether duloxetine acts through this certain brain region. Additionally, we used fiber photometry to measure the effect of duloxetine on the activity of glutamatergic neurons and the concentration of serotonin in ACC.

## Materials and methods

### Animals and groups

#### Animals

Our experiments were performed under the approval of the IACUC at ShanghaiTech University. C57BL/6J male mice (Vital River Shanghai China) aged 8–10 weeks were utilized in all experiments. Mice were housed up to five per cage with free access to food and water and maintained a 12 h light/dark cycle (lights on from 7 a.m. to 7 p.m.) at a stable temperature ranging from 22 to 25°C. Throughout the whole experiment, 90 mice were used in total.

#### Groups

In fiber photometry test, after the injection of rAAV-CaMKIIa-GCamp6s or rAAV-hSyn-5HT3.5 and a 14-day recovery, C57BL/6J male mice were divided into two groups: duloxetine and saline, where duloxetine group received intraperitoneal duloxetine injection. In contrast, the saline group received an intraperitoneal saline injection (Table 1).

**TABLE 1:** Outline of experimental design.

Experiment design	Groups and treatment
**Fiber photometry**	
Virus injection in anterior cingulate cortex (ACC) before fiber plantation: rAAV-CaMKIIa-Gcamp6s rAAV-hSyn-5HT3.5 Recording after a 14-day recovery.	Duloxetine: Intraperitoneal injection of duloxetine while recording Saline: intraperitoneal injection of saline while recording.
**Systemic injection of duloxetine**	
After complete Freund’s adjuvant (CFA) modeling, von Frey test was taken in 1, 5, 10, 15, and 20 days after CFA (saline) injection. Elevated plus maze test (EPM) and novelty-suppressed feeding test (NSF) were taken at day 21 and 22. After treatment, repeated behavioral tests was taken in Duloxetine group an Saline group.	CFA: mice underwent CFA injection in the left hind paw. SHAM: mice underwent saline injection in the left hind paw. Duloxetine: CFA mice that showed anxiety-lie behavior 21 days after modeling. A total of 3-days i.p. injection of duloxetine. Saline: CFA mice that showed anxiety-lie behavior 21 days after modeling. A total of 3-days i.p. injection of saline.
**Local infusion of duloxetine in ACC**	
Cannulae were planted in mice 3 days before CFA modeling, identical behavioral tests was conducted before and after treatment as systemic injection.	CFA: mice underwent CFA injection in the left hind paw. SHAM: mice underwent saline injection in the left hind paw. Duloxetine: CFA mice that showed anxiety-lie behavior 21 days after modeling. A total of 3-days ACC infusion of duloxetine. Vehicle: CFA mice that showed anxiety-lie behavior 21 days after modeling. A total of 3-days ACC infusion of 10% dimethyl sulfoxide (DMSO).

In systemic duloxetine injection, mice that underwent CFA injection in the left hind paw are defined as the CFA group, while the mice receiving a saline injection in the left hind paw are defined as the SHAM group. After the modeling and baseline behavioral tests, CFA mice were divided into two groups: the duloxetine group and the saline group, where they received their corresponding intraperitoneal injections for 3 days (Table 1).

In ACC local infusion, similar to the grouping logic in systemic duloxetine injection, mice were divided into CFA and SHAM groups after CFA and saline injections in the left hind paw. CFA mice are divided into duloxetine and vehicle groups; the duloxetine group received intracerebral duloxetine [dissolved in 10% dimethyl sulfoxide (DMSO)] injection, while the vehicle group received intracerebral 10% DMSO (Table 1).

### Animal models

#### Inflammatory pain model

Inflammatory pain was induced by the injection of CFA (20 μl) into the left hind paw of the mouse (Wang et al., 2015; Zhu et al., 2021). Matched amount of saline was injected into the same place as the SHAM group. On the 12th day after the first injection, the second dose of CFA/saline was injected into the same site to prolong the effect of the model (Jin et al., 2020).

### Behavioral tests

#### Von Frey filament test

The mechanical withdrawal threshold was measured by the von Frey filament (Stoelting America, Illinois, IL, USA) using an up-down method (Chaplan et al., 1994). Mice were placed on an elevated metal mesh board and under the cover of transparent plastic chambers (9 cm × 9 cm × 4.5 cm). This test was only performed in the light cycle. Before the sequencing tests, the mice should be habituated for 3 days. On the first day of habituation, the mice were put on the board until they fell asleep (about 60–90 min), while on the next 2 days, they were applied two rounds of filament (1 g) probing to the left hind paw (where CFA was injected). During the formal tests, von Frey filaments (bending force ranging from 0.008 to 1.4 g) were applied five times (3 s for each stimulus) to the test site mentioned above. Sudden paw withdrawal and paw licking were considered positive responses. The smallest bending force of the von Frey filament that evokes positive responses with over 50% occurrence frequency is regarded as the mechanical threshold of the mice.

#### Elevated plus maze test

Anxiety-like behavior was tested by the elevated plus maze test (EPM) with a generally identical procedure to the previous study (Ni et al., 2022). Mice were placed in a cross-shaped, elevated maze with an open arm and a closed arm (50 cm above the ground, open arm: 27 cm × 7 cm; close arm: 27 cm 7 cm × 15 cm). They were placed on the central platform facing the open arm, and their behavior was recorded for 300 s. The time spent in the open arm (the time of mice sticking at least half of their body out of the open arm is considered in the open arm) was collected. The result was presented as the ratio of time in the open arm to time in the closed arm.

#### Novelty-suppressed feeding test

The anxiodepressive-like behavior was confirmed by the novelty-suppressed feeding test (NSF) (Marcinkiewcz et al., 2016). The testing apparatus of NSF consists of an open field box (25 cm × 25 cm × 25 cm) covered with the identical bedding used in the home cage. A piece of fruit loops (Kellogg’s, Michigan, MICH, USA) was placed on a circular white filter paper in the middle of the open field. After 24 h of being singly caged and food deprivation, the mice were placed in the corner of the open field. The latency to have the first bite of the food was recorded, up to 10 min. After the test, the mice were moved back to the home cage with free access to the fruit loops to measure the food intake for 5 min. The weight before and after home cage feeding was recorded.

### Drug administration

Duloxetine (SNRIs, duloxetine hydrochloride enteric capsules, Cymbalta ^®^ Eli Lilly; Beyotime Biotechnology, Shanghai, China) was dissolved in 0.9% saline or 10% DMSO in different concentrations as follows: 20 mg/kg for intraperitoneal injection (i.p.) and 0.25 ml of duloxetine was injected; 200 mg/kg for intracerebral injection (i.c.) and 1 μl of duloxetine was injected. During behavioral tests, duloxetine was given around 1 p.m. for 3 days.

### Surgery

rAAV-CaMKIIa-GCamp6s (≥ 5.00E + 12 vg/ml) and rAAV-hSyn-5HT3.5 (≥ 5.00E + 12 vg/ml) were purchased from BrainVTA (Wuhan, China). Optical fiber and cannulae were purchased from RWD Life Science Co., LTD., (Shenzhen, China). The procedures were generally identical to the previous study (Li et al., 2020). Mice were anesthetized with isoflurane (4% for induction and 1.5% for maintenance). Then, they were placed in a stereotactic apparatus to adjust the skulls parallel to the reference panel. A total of 200 nl of the virus were injected into unilateral ACC gradually in 5 min (Bregma: −0.97 mmAP, ±0.25 mmML, −1.5 mmDV) (Paxinos and Franklin, 2019) by using a micro syringe injector followed by a 10-min interval for viral particle diffusion before planting the optical fiber to the target site (Bregma: −0.97 mmAP, ±0.25 mmML, −1.3 mmDV). The cannula implantation steps were identical to what was mentioned, excluding the AAV injection process.

### Fiber photometry

After 2 weeks of recovery, the mice were ready for the Ca^2+^ signal recording procedure. The procedure was generally identical to previous studies (Li et al., 2020; Zhu et al., 2021). In brief, Ca^2+^ signals were obtained from a fiber photometry system. The analog voltage signals were digitalized at 100 Hz and were recorded by a Power 1401 digitizer and Spike2 software (CED, Cambridge, UK). The laser power was adjusted to 20–40 μW. The data we retrieved were exported to MATLAB R2020a mat files (The MathWorks, Massachusetts, MA, USA). The first step was to correct the bleaching of the signal. An approximately 300-s-long section from the incipient and ending stage was selected to fit a curve through these two phases. We derived the values of Ca^2+^ signal changes (ΔF/F) by calculating (F–F0)/F0, which were presented with average plots with several curves representing the signal changes. Specifically, F0 represents the mean of the signal during a 500 s baseline. To obtain the value for statistics, the mean of ΔF/F values 300 s before drug injection is calculated as the control value and the mean of ΔF/F values during the first 3,500 s after injection as the treatment value.

The instrument and procedure of neurotransmitter sensor recording resemble Ca^2+^ signal recording. The only difference is that we obtained the mean of ΔF/F values during the first 5,000 s after injection as the treatment value.

### Histology

The mice were anesthetized by the intraperitoneal injection of tribromoethanol (mass fraction: 2.5%, i.p.) followed by saline perfusion through the heart and then fixation by 4% paraformaldehyde (PFA) after blood was drained. The brain was removed the next day and transferred to 30% sucrose in 0.1 M PBS, pH 7.4, for 24 h. We used cryostat (Leica CM3050S) to obtain coronal sections (40 μm). The slides were washed with 0.1 M PBS, pH 7.4. We performed 4’,6-diamidine-2-phenylindole (DAPI) staining to identify the cell bodies and 10% of glycerin to seal the slides. Fluorescent images were scanned *via* an Olympus VS 120 microscope.

### Statistical analysis

All statistical analyses were completed in GraphPad Prism 9 (GraphPad Software, California, CA, USA) or MATLAB R2020b programs (The MathWorks, Massachusetts, MA, USA). We used paired or unpaired *t*-tests for different needs accordingly. Mann–Whitney test was used if the data had significantly different variances. Differences between groups were judged to be statistically significant when *p*-values is < 0.05. Asterisks denote statistical significance *p < 0.05; ^**^p < 0.01; ^***^p < 0.001; ^****^p < 0.0001.

## Results

### Duloxetine increases serotonin concentration while deactivating glutamatergic neurons in anterior cingulate cortex

**FIGURE 1:**
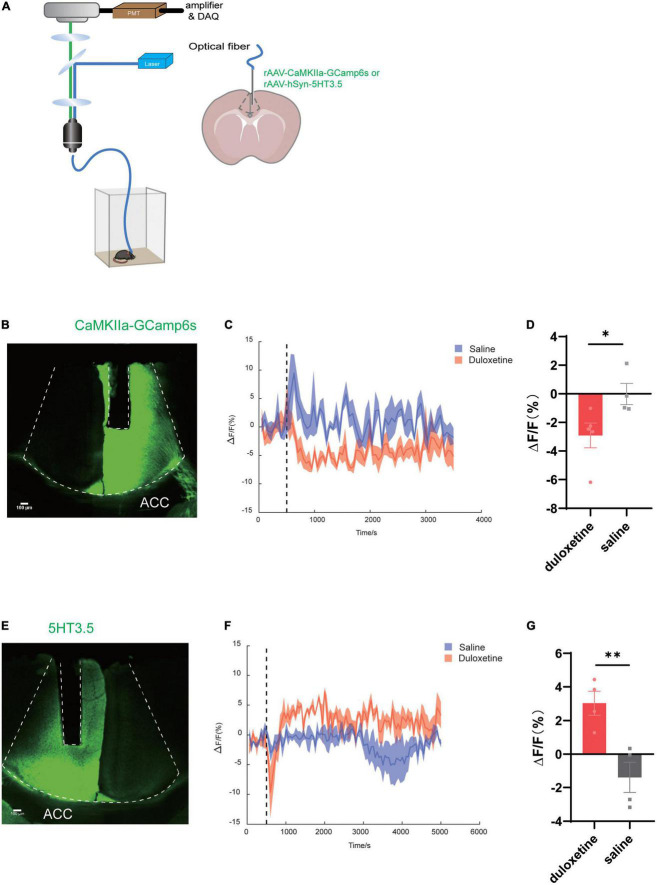
Duloxetine increases serotonin concentration while deactivating glutamatergic neurons in anterior cingulate cortex (ACC). **(A)** Schematic diagram of the experimental procedure for fiber photometry of 5-HT sensor fluorescence signals and Ca^2+^ signal recording of CaMKIIa-Gcamp6s of mice ACC. **(B)** Image of CaMKIIa-Gcamp6s and the fiber channel in ACC. **(C)** Fluorescence changes of CaMKIIa-Gcamp6s in ACC after the intraperitoneal injection of duloxetine. **(D)** Average ΔF/F after saline and duloxetine injection (Duloxetine *n* = 5, saline *n* = 4, *p* = 0.0434, unpaired *t*-test). **(E)** Image of the 5-HT sensor and the fiber channel in ACC. **(F)** Fluorescence changes of the 5-HT sensor in ACC after the intraperitoneal injection of duloxetine. **(G)** Average ΔF/F after saline and duloxetine injection (*n* = 4, *p* = 0.0083, unpaired *t*-test).

To discover the target region of duloxetine and its underlying mechanism, we planted optic fiber after injecting rAAV-CaMKIIa-Gcamp6s into either the right or left ACC of C57BL/6J male mice to measure the activity of glutamatergic neurons. We also injected rAAV-hSyn-5HT3.5 into the same site of another batch of mice to measure local serotonin levels (Figures 1A,B,E).

We found that duloxetine had increased serotonin concentration in ACC compared with saline (*n* = 4, *p* = 0.0083, unpaired *t*-test; Figures 1F,G) based on the data recorded by fiber photometry, while it decreased the activity of glutamatergic neurons (duloxetine *n* = 5, saline *n* = 4, *p* = 0.0434, unpaired *t*-test; Figures 1C,D) based on the result of Ca^2+^ signal recording.

### Systemic duloxetine improved mechanical withdrawal threshold and anxiety-like behaviors in mice with chronic inflammatory pain

**FIGURE 2:**
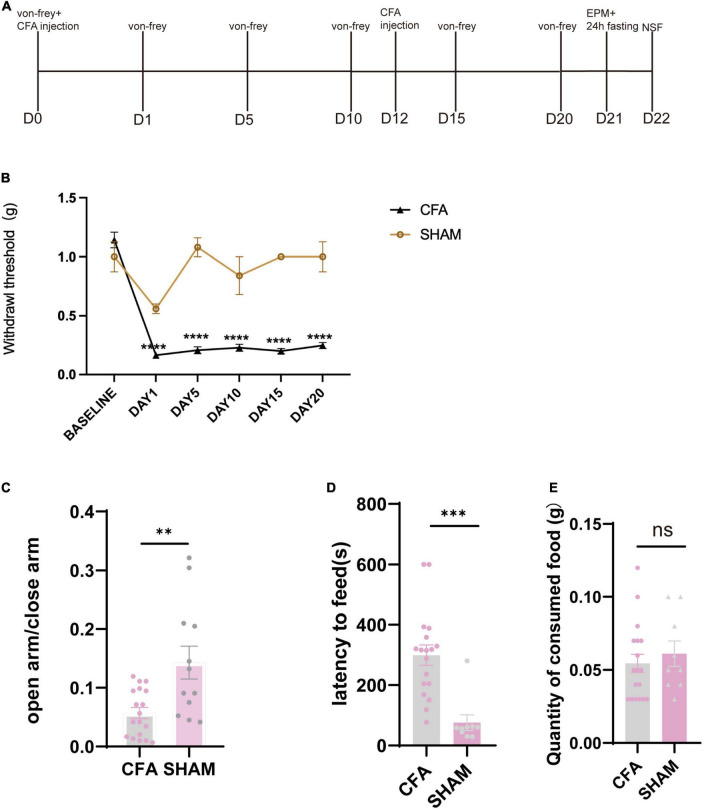
Mice showed anxiety-like behavior after the chronic inflammatory pain model. **(A)** Timeline of CFA injection and subsequent behavioral tests. **(B)** Withdrawal threshold of CFA and SHAM groups during the modeling process (CFA: *n* = 24, SHAM: *n* = 12 baseline *p* = 0.4690 days 1–20: *p* < 0.0001, Mann–Whitney test). **(C)** The ratio of time in the open arm and time in the closed arm of CFA and SHAM groups 21 days after the first CFA injection (CFA: *n* = 18, SHAM: *n* = 12, *p* = 0.0023, unpaired *t*-test). **(D)** Latency to feed of CFA and SHAM groups (CFA: *n* = 18, SHAM: *n* = 9, *p* = 0.0002, unpaired *t*-test). **(E)** Quantity of food consumed in home cage feeding test (CFA: *n* = 18, SHAM *n* = 9, *p* = 0.5408, unpaired *t*-test).

To examine duloxetine’s therapeutic effect on pain-induced anxiety, we used CFA (saline was injected in the SHAM group) model to induce inflammatory pain in mice. We replenished another injection on day 12 from the first shot to maintain the algetic effect (Figure 2A). We used von Frey filament to measure the mechanical withdrawal threshold at baseline, days 1, 5, 10, 15, and 20 to ensure that CFA mice have a significantly lower mechanical withdrawal threshold than the SHAM group (CFA: *n* = 24, SHAM: *n* = 12 baseline *p* = 0.4690 days 1–20: *p* < 0.0001, Mann–Whitney test; Figure 2B). On days 21 and 22, EPM tests were used to identify the CFA mice that have successfully induced anxiety-like behaviors. The result indicated that mice expressed anxiety-like behaviors 21 days after CFA injection. To be specific, CFA mice spent significantly less time in the open arm in the EPM test (CFA: *n* = 18, SHAM: *n* = 12, *p* = 0.0023, unpaired *t*-test; Figure 2C), while the latency to feed is significantly longer than the SHAM group (CFA: *n* = 18, SHAM: *n* = 9, *p* = 0.0002, unpaired *t*-test; Figures 2D,E).

**FIGURE 3:**
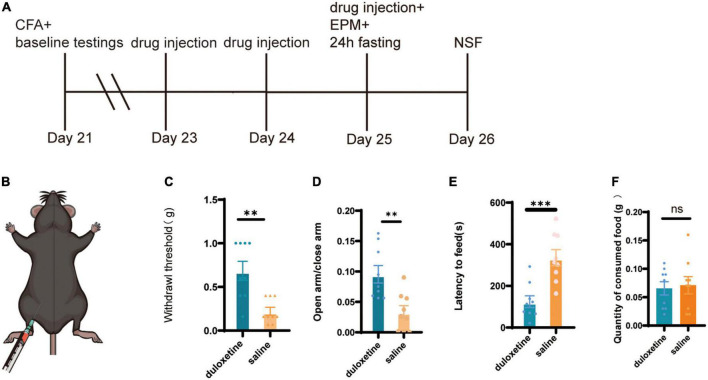
Systemic duloxetine improved mechanical withdrawal threshold and anxiety-like behaviors in mice with chronic inflammatory pain. **(A)** Timeline of duloxetine injection and subsequent behavioral tests. **(B)** Intraperitoneal injection of duloxetine. **(C)** Withdrawal threshold of duloxetine and saline group after a 3-day injection (*n* = 9, *p* = 0.0012, unpaired *t*-test). **(D)** The ratio of time in the open arm to closed arm of duloxetine and saline groups (*n* = 9, *p* = 0.0012, unpaired *t*-test). **(E)** Latency to feed of duloxetine and saline groups (*n* = 9, *p* = 0.0004, unpaired *t*-test). **(F)** Quantity of consumed food in home cage feeding test (*n* = 9, *p* = 0.7751 unpaired *t*-test).

After the baseline behavior tests, mice with anxiety-like behavior were selected and divided into two groups: the duloxetine group and the saline group. The duloxetine group received duloxetine (20 mg/kg, i.p.) for 3 days, while saline was injected into the saline group (Figures 3A,B). Then, identical tests were performed to measure the therapeutic effect of duloxetine. Von Frey filament showed that the mechanical withdrawal threshold of the duloxetine group is significantly higher than the saline group (*n* = 9, *p* = 0.0029, Mann–Whitney test; Figure 3C). In the EPM test, the duloxetine group spent significantly more time in the open arm (*n* = 9, *p* = 0.0012, unpaired *t*-test; Figure 3D), while the latency to feed was significantly shorter than the saline group in the NSF test (*n* = 9, *p* = 0.0004, unpaired *t*-test; Figures 3E,F). Systemic duloxetine improved mechanical withdrawal threshold and anxiety-like behaviors in mice with chronic inflammatory pain.

### Intra-anterior cingulate cortex duloxetine improved mechanical withdrawal threshold but did not affect anxiety-like behaviors in mice with chronic inflammatory pain

**FIGURE 4:**
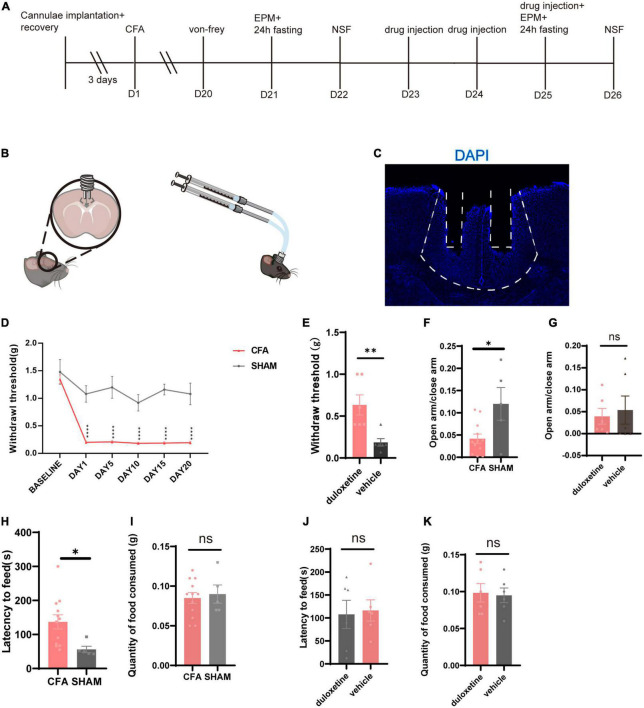
Intra-anterior cingulate cortex (ACC) duloxetine improved mechanical withdrawal threshold but had no effect on anxiety-like behaviors in mice with chronic inflammatory pain. **(A)** Timeline of cannula implantation, CFA injection, behavioral tests, and drug infusion. **(B)** Cannula implantation and local infusion. **(C)** Representative brain slice of cannulae implantation in ACC. **(D)** Withdrawal threshold of CFA and SHAM groups during the modeling process (CFA: *n* = 26, SHAM: *n* = 5 baseline *p* = 0.7398 days 1–20: *p* < 0.0001, Mann–Whitney test). **(E)** Withdrawal threshold of duloxetine and saline group after a 3-day local infusion (*n* = 6, *p* = 0.0087, Mann–Whitney test). **(F)** The ratio of time in the open arm and time in the closed arm of CFA and SHAM groups 21 days after the first CFA injection (CFA: *n* = 12, SHAM: *n* = 5, *p* = 0.0131, unpaired *t*-test). **(G)** The ratio of time in the open arm and time in the close arm of duloxetine and vehicle groups after local infusion (*n* = 6, *p* = 0.7064, unpaired *t*-test). **(H)** Latency to feed of CFA and SHAM groups (CFA: *n* = 12, SHAM: *n* = 5, *p* = 0.0290, unpaired *t*-test) and **(I)** quantity of food consumed in a home cage feeding test 21 days after the first CFA injection (CFA: *n* = 12, SHAM *n* = 5, *p* = 0.8291, unpaired *t*-test). **(J)** Latency to feed of duloxetine and vehicle group (*n* = 6, *p* = 0.7020, unpaired *t*-test) and **(K)** quantity of food consumed in home cage feeding test after a 3-day local infusion (*n* = 6, *p* = 0.8387, unpaired *t*-test).

We planted cannulae in bilateral ACC in mice (Figures 4B,C) and used identical procedures of modeling and screening (Figure 4A). We picked CFA (Figure 4D) mice that have successfully induced anxiety-like behaviors (EPM: CFA: *n* = 12, SHAM: *n* = 5, *p* = 0.0131, unpaired *t*-test; Figure 4F; NSF: CFA: *n* = 12, SHAM: *n* = 5, *p* = 0.0290, unpaired *t*-test; Figures 4H,I) and divided into duloxetine and vehicle groups. Like systemic injection, we consecutively injected duloxetine (10% DMSO for the vehicle group) for 3 days before using behavior tests to determine the effect of local infusion in ACC. We found out that the mechanical withdrawal threshold of duloxetine was significantly higher than those of the saline group (*n* = 6, *p* = 0.0087, Mann–Whitney test; Figure 4E). However, the EPM test (*n* = 6, *p* = 0.7064, unpaired *t*-test; Figure 4G) and the NSF test (*n* = 6, *p* = 0.8291, unpaired *t*-test; Figures 4J,K) showed no difference between the two groups. In conclusion, intra-ACC duloxetine improved the mechanical withdrawal threshold but did not affect anxiety-like behaviors in mice with chronic inflammatory pain.

## Discussion

In this study, we discovered the analgesic and anxiolytic effects of duloxetine in mice with chronic inflammatory pain-induced anxiety. Second, we found the analgesic but not the anxiolytic effect of duloxetine when locally injected into ACC. Furthermore, we unveiled that duloxetine increased serotonin concentration while decreasing the activity of glutamatergic neurons in ACC.

Duloxetine and other SNRIs are considered the first-line therapeutic in clinical practice for neuropathic pain (Finnerup et al., 2015). Besides, it is also one of the antidepressants used to treat generalized anxiety disorder (Kupfer et al., 2012; Slee et al., 2019). Based on these two therapeutic effects of duloxetine, we took an assumption. We verified that it has analgesic and anxiolytic effects in chronic pain-induced anxiety by the systemic injection of duloxetine in CFA mice. Ito et al. (2018) have found that duloxetine attenuated the mechanical hyperalgesia induced by spinal nerve ligation which coincided with our finding. However, the model we used, CFA, is not a typical neuropathic pain model but an inflammatory pain. The fact that duloxetine could alleviate inflammatory pain is an inspiration that duloxetine could facilitate the treatment of not only neuropathic pain but other categories of them. There were clinical trials of duloxetine’s pain reduction effect in knee osteoarthritis (Frakes et al., 2011; Enteshari-Moghaddam et al., 2019). Above all, duloxetine showed a promising potential to alleviate not only neuropathic pain but also inflammatory pain.

Although duloxetine is clinically used for treating pain, the mechanism of its analgesic effect remains unclear. Through our discoveries, we could first glance at its target brain region and its modulation properties with different neurotransmitters. First, we chose ACC as a potential target due to its reported connection and role in pain, stress, and chronic pain-induced anxiety (Shackman et al., 2011; Bliss et al., 2016). The current study did not verify that duloxetine could upregulate 5-HT1A receptors in ACC; therefore, more research is needed to confirm. However, as one of the SNRIs, it indeed increases the serotonin concentration in ACC to inhibit the activity of glutamatergic neurons, as is proved by our fiber photometry result. In addition, intra-ACC injection of duloxetine attenuated the mechanic withdrawal threshold in CFA mice. These results could verify our previous assumption on the mechanism of duloxetine in ACC with the help of the findings from other studies mentioned in our section “Introduction” (Marcinkiewcz et al., 2016; Tian et al., 2017; Paredes et al., 2019).

Furthermore, our data revealed that the intra-ACC injection of duloxetine could not alleviate the anxiety-like behaviors in CFA mice, whereas systemic duloxetine could alleviate, suggesting that ACC is not the target brain region for duloxetine’s anxiolytic effect. However, ACC still play a vital role in chronic pain-induced anxiety. Still, it urges that the anxiolytic effect of duloxetine is due to its interaction with certain neurotransmitters or receptors in other brain regions that are possibly upstream or downstream of a neuronal circuit involving ACC. Zhuo (2016) has proposed that ACC may act as a higher structure that integrates anxiety signaling through the projection from the amygdala, which is also involved in anxiety and fear. Therefore, further studies should focus on anxiolytic effect of duloxetine and potential mechanism in the amygdala. Last, Barthas et al. (2015) found that repeated optogenetic stimulation in pyramidal neurons in ACC induced anxiodepressive-like behaviors in mice. Besides, the ablation of ACC after the chronic pain model could prevent the anxiodepressive-like behavior taken 6 weeks after surgery. Such results coincided with our theory above and provided us with a potential usage of duloxetine which could inhibit the glutamatergic activity in ACC. The early intervention of duloxetine might prevent the induction of anxiety due to chronic pain. This assumption, along with the potential anxiolytic mechanism of duloxetine, is the future direction.

### Limitations and future directions

This study only used duloxetine with fixed concentration, and we only tested the mechanical allodynia. The behavioral test for heat hyperalgesia is missed. Besides, we did not locate the brain region where duloxetine could achieve its anxiolytic effect. Therefore, we intended to study further the anxiolytic effect of duloxetine and its mechanism in mice with chronic pain-induced anxiety.

We used DMSO as the vehicle for duloxetine in local infusion into ACC. It is reported in previous studies that the intraperitoneal injection of 10% DMSO led to significant motor impairment (Matias et al., 2018). Furthermore, another study discovered that local infusion to dorsal periaqueductal gray could increase the exploratory activity in the EPM test (Matheus et al., 1997). Further verification should be done to rule out the potential interference of DMSO which might cause the result in our study.

## Conclusion

This study revealed the analgesic and anxiolytic effects of duloxetine in mice with chronic inflammatory pain. However, duloxetine lost its anxiolytic effect when locally injected into ACC. Moreover, we unveiled that duloxetine increased serotonin concentration while decreasing the activity of glutamatergic neurons in ACC.

## Data availability statement

The original contributions presented in this study are included in the article/supplementary material, further inquiries can be directed to the corresponding author.

## Ethics statement

This animal study was reviewed and approved by the ShanghaiTech University.

## Author contributions

CL designed the study, conducted the experiments, analyzed the data, and wrote the article. KN analyzed the data and revised the article. MQ and KY assisted in part of the behavioral tests. JL and YL helped to design the study and revised the article. All authors contributed to the article and approved the submitted version.

## Abbreviations

ACC: anterior cingulate cortex
CFA: complete Freund’s adjuvant
pre-LTP: presynaptic long-term potentiation
DMSO: dimethyl sulfoxide
EPM: elevated plus maze test
NMDA: N-methyl-D-aspartate
NSF: novelty-suppressed feeding test
PFA: paraformaldehyde
SNI: spared nerve injury
SNRIs: serotonin–norepinephrine reuptake inhibitors.
